# Anthropometric measurements and their association with endothelial function and arterial stiffness of eutrophic individuals and with overweight

**DOI:** 10.20945/2359-3997000000617

**Published:** 2023-05-29

**Authors:** Ariélen Ferigollo, Diego Chemello, Tábata Pereira Pavão, Marco Aurélio Lumertz Saffi, Carolina dos Santos Stein, Rafael Noal Moresco, Lucas Silva de Souza, Carlos Heitor Cunha Moreira, Luis Ulisses Signori, Patrícia Chagas

**Affiliations:** 1 Universidade Federal de Santa Maria Pós-graduação em Gerontologia Santa Maria RS Brasil Programa de Pós-graduação em Gerontologia, Universidade Federal de Santa Maria (UFSM), Santa Maria, RS, Brasil; 2 Universidade Federal de Santa Maria Departamento de Clínica Médica Santa Maria RS Brasil Departamento de Clínica Médica, Universidade Federal de Santa Maria (UFSM), Santa Maria, RS, Brasil; 3 Hospital de Clínicas de Porto Alegre Porto Alegre RS Brasil Hospital de Clínicas de Porto Alegre (HCPA), Porto Alegre, RS, Brasil; 4 Universidade Federal de Santa Maria Programa de Pós-graduação em Ciências Farmacêuticas Santa Maria RS Brasil Programa de Pós-graduação em Ciências Farmacêuticas, Universidade Federal de Santa Maria (UFSM), Santa Maria, RS, Brasil; 5 Universidade Federal de Santa Maria Graduação em Medicina Santa Maria RS Brasil Graduação em Medicina, Universidade Federal de Santa Maria, Santa Maria, RS, Brasil; 6 Universidade Federal de Santa Maria Faculdade de Odontologia Departamento de Estomatologia Santa Maria RS Brasil Departamento de Estomatologia, Divisão de Periodontologia, Faculdade de Odontologia, Universidade Federal de Santa Maria (UFSM), Santa Maria, RS, Brasil; 7 Universidade Federal de Santa Maria Departamento de Fisioterapia e Reabilitação Programa de Pós-graduação em Ciências do Movimento e Reabilitação Santa Maria RS Brasil Departamento de Fisioterapia e Reabilitação, Programa de Pós-graduação em Ciências do Movimento e Reabilitação, Universidade Federal de Santa Maria (UFSM), Santa Maria, RS, Brasil; 8 Universidade Federal de Santa Maria Departamento de Alimentação e Nutrição Programa de Pós-graduação em Gerontologia Santa Maria RS Brasil Departamento de Alimentação e Nutrição, Programa de Pós-graduação em Gerontologia, Universidade Federal de Santa Maria (UFSM), Santa Maria, RS, Brasil

**Keywords:** Anthropometry, nutritional assessment, vascular endothelium, vascular stiffness, cardiovascular diseases

## Abstract

**Objective:**

The objective of the study was to assess the association of anthropometric measurements with endothelial function and arterial stiffness of eutrophic individuals and with overweight.

**Subjects and methods:**

A cross-sectional study was carried out with individuals with body mass index (BMI) between 18.5 kg/m² and < 30 kg/m², low to intermediate global cardiovascular risk scores, and aged ≥ 18 and < 60 years. We assessed the sociodemographic data, anthropometric variables (body weight, height, circumferences of the waist [WC], neck [NC], hip [HC], sagittal abdominal diameter [SAD], [BMI], waist-to-hip ratio [WHR], and waist-to-height ratio [WHtR]), biochemical parameters (lipid profile and nitric oxide), endothelial function (flow-mediated dilation [FMD], by ultrasound), and arterial stiffness (pulse wave velocity [PWV] and the amplification index [AIx@75] by oscillometry). Thirty-six individuals were included, 18 eutrophic and 18 with overweight, with a mean age of 37.5 ± 10.2 years, mostly at low cardiovascular risk (86.1%), female (80.6%), single (52.8%), employed with formal contracts (44.4%), and with over twelve years of education (88.9%).

**Results:**

The PWV presented positive and moderate correlation with the WC (r = 0.584; P = 0.001), WHR (r = 0.513; P = 0.001), and WHtR (r = 0.590; P = 0.001), and positive and low correlation with the NC (r = 0.372; P = 0.013) and SAD (r = 0.356; P = 0.033). Moreover, no anthropometric parameter presented a correlation with the AIx@75 or the FMD percentage in the total sample.

**Conclusion:**

Our findings show that in eutrophic individuals and with overweight the WC, WHR, WHtR, SAD, and NC were positively correlated with the PWV but not to the endothelial function in the overall sample. These are hypothesis-generating findings and they should be replicated in other studies.

## INTRODUCTION

In recent decades, the prevalence of excess body weight has been increasing rapidly in both developed and developing countries (1). In Brazil, 53.8% of the population was affected by overweight, and 18.9% with obesity up to 2016 (2). The increase in body weight, especially excess abdominal and visceral fat, is recognized as an independent risk factor for developing various health problems such as cardiovascular diseases (CVDs), besides influencing the increase in mortality (3,4).

The impact that excess weight has on cardiovascular function encompasses various complex factors and mechanisms, such as the inflammatory state induced by the excess fatty tissue. Inflammation seems to be one of the main causes that lead to the activation of mechanisms connected to vascular lesions, endothelial dysfunction, and arterial stiffness. Arterial stiffness is considered to be one of the precursors of atherosclerotic lesions and is strongly associated with the development of endothelial dysfunction (5,6).

Endothelial function and arterial stiffness may be assessed by various non-invasive and replicable techniques such as the brachial artery flow-mediated dilation (FMD) and the automated oscillometric measurement, respectively (7,8). FMD assesses the image of the brachial artery in response to reactive hyperemia, inducing the elevation of local flow and the expected endothelium-dependent dilation (9). In turn, the arterial stiffness assessment by analyzing the brachial artery area and vascular elasticity allows the detection of structural and functional modifications of the arteries, with pulse wave velocity (PWV) and the amplification index (AIx@75) being the measures most commonly used (10).

Some studies have evinced the association between body fat assessment measurements with the occurrence of subclinical atherosclerosis and with the CVDs (11,12).

Knowing the negative impact that CVDs may have on health, there is a need to improve more and more the early identification of individuals at cardiovascular risk. The works that describe the relationship of anthropometric indicators with endothelial function and arterial stiffness parameters in healthy individuals are scarce and with distinct results (13-22). Therefore, the present research sought to investigate which anthropometric measurements better correlate with the endothelial function and the arterial stiffness parameters in eutrophic individuals and with overweight.

## SUBJECTS AND METHODS

### Study design and subjects

This is a cross-sectional study. The convenience sampling consisted of adults aged between 18 and 59, eutrophic or with overweight, and who had low to intermediate cardiovascular risk as per the Global Risk Score (GRS) (23). The collections occurred from February to July 2019 at the Laboratory of Autonomic Diseases of the *Instituto do Coração* (ICor), in Santa Maria, Brazil. The cardiovascular risk assessment was performed by a cardiologist certified by the Brazilian Society of Cardiology (D.C.). The cardiovascular risk was established as low, intermediate, high, or very high according to the Global Risk Score (GRS). Individuals with intermediate risk were defined as those with 5-20% of having a cardiovascular outcome in 10 years (men) or with 5%-10% of risk (women). Low risk was defined as individuals with less than 5% chance of having a cardiovascular outcome in 10 years (24,25).

Exclusion criteria were: individuals with body mass index (BMI) lower than 18.5 kg/m² or equal or higher than 30 kg/m²; coronary artery disease; heart failure; persistent or permanent atrial fibrillation; liver cirrhosis; serious chronic obstructive pulmonary disease; peripheral artery disease; neurological disease; infectious diseases that were active or had been treated in the past 30 days; type 1 or type 2 diabetes mellitus; smokers; and individuals who had been hospitalized in the two months before the research.

The checking of the fulfillment of the inclusion and exclusion criteria, as well as the cardiovascular risk assessment of the volunteers for the study, was performed by a cardiologist of the research team (D.C.).

#### Sociodemographic variables

The sociodemographic variables (gender, age, marital status, profession, self-reported race, and education level) were assessed through a structured questionnaire.

### Anthropometric assessments

The anthropometric measurements were taken with the subjects wearing only light clothes. For obtaining the body weight and height, a calibrated anthropometric digital scale (Filizola®, São Paulo, Brazil) was used, with the subjects in orthostatic position, arms hanging by the sides of the body, and feet bare and united (24). Waist circumference was measured at the thinnest point between the last rib and the iliac crest (2,25). Hip circumference was assessed at the region with the largest perimeter of the hip (24). Neck circumference was measured at the midpoint of the neck in women, and just below the laryngeal prominence in men (26,27). The sagittal abdominal diameter was assessed using a segmometer (Cescorf®, Porto Alegre, Brazil) with a mobile rod and 0.1 subdivision. The mobile rod of the caliper was closed until the region immediately below the belly button, with the patient in an orthostatic position and arms crossed over the chest (24).

The BMI was calculated by dividing the weight (kg) by the squared height (m) and classified as follows: underweight ≤ 18.5 kg/m²; eutrophic 18.5-24.9 kg/m²; with overweight 25.0-29.9 kg/m²; and with obesity ≥ 30.0 kg/m² (28). The waist-to-hip ratio was obtained by dividing the circumference of the waist by that of the hip (28), while the waist-to-height ratio was calculated by dividing the circumference of the waist by the height (29).

### Assessment of endothelial function and arterial stiffness

FMD is the non-invasive method used to assess endothelial function, which was evaluated as described in the guidelines of the American College of Cardiology (30,31). The brachial artery images were acquired using a high-resolution ultrasound machine (PureWare HD15 by Phillips® with a linear transducer with a frequency of 7 Hz to 12 Hz). The region analyzed was above the antecubital fossa of the right arm, on a longitudinal plane, with the patient lying down in dorsal decubitus. The cuff was positioned around the forearm and insufflated 50 mmHg above the systolic arterial pressure, maintaining the ischemia for five minutes. After this period, the cuff pressure was completely released, generating vasodilation (reactive hyperemia). The arterial image was obtained one minute after the deflation of the cuff. All the images were obtained at the beginning of the R wave of the electrocardiogram, coinciding with the final period of the diastole. To determine the artery diameter, at least three measurements were taken and considered valid when the variation among them was smaller than 10%. The vasodilation response was expressed as a percentage alteration in the diameter (FMD%). The assessments were carried out in a calm environment with controlled temperature (between 22 °C and 25 °C) and low luminosity. All volunteers received previous guidance to abstain from beverages containing caffeine, red fruits, cocoa, or dark chocolate, supplements with antioxidant activity, and alcoholic beverages for 72 hours before the test. They were also advised to keep a regular diet, avoid consuming fat-rich products, and not undergo physical activity on the day of the assessment. The participants attended the study site on a six-hour fast.

The arterial stiffness was assessed using the oscillometric device Dyna-MAPA AOP, (Cardios®, São Paulo, Brazil). This device allows measuring arterial stiffness parameters using an oscillographic method on the brachial artery, having been previously validated (32). We obtained the values regarding the central systolic arterial pressure (SAP) and the central diastolic arterial pressure (DAP), mean arterial pressure (MAP), cardiac output, total vascular resistance, amplification index (AIx@75), and pulse wave velocity (PWV) (10). Three measurements were obtained, and the means were calculated for analysis. The tests were carried out before endothelial function, on the left arm, and with the individual seated.

### Biochemical parameters

Blood samples were collected from the individuals after at least 6 h fasting, into Vacutainer® (BD Diagnostics, New Jersey, USA) tubes without anticoagulants. Serum was obtained after centrifugation of blood at 2500 × *g* for 15 min. The lipid profile was obtained through commercial kits with colorimetric methodologies (Bioclin®, Belo Horizonte, Brazil), except for the LDL-cholesterol, which was estimated using the Friedewald formula (33). The nitric oxide metabolites nitrite and nitrate (NOx) concentrations were measured using the modified Griess method (34), in the automated system BS 380® (Mindray, Shenzhen, China).

### Statistical analysis

The comparison between means was analyzed with Student's T-Test, while the association among categorical variables with Pearson's Qui-Square Test and Fisher's Exact Test. Pearson's Test was used for the correlations of the quantitative variables. We deemed significant the analyses with P< 0.05. The results of such correlations were classified as described by Mukaka (35), considering correlations with r = 0.00 to 0.30 as insignificant, r = 0.30 to 0.50 as low, r = 0.50 to 0.70 as moderate, r = 0.70 to 0.90 as high, and 0.90 to 1.00 as very high.

The sample size was estimated using the Power and Sample Size Program through the T-Test. A sample of 18 individuals per group is needed to show a significant mean difference of 0.90 in the FMD% among eutrophic, individuals with overweight, and with obesity groups as assessed by the BMI, assuming a standard deviation of 0.73, a 20% loss rate, a 90% power, and a 5% significance level (36).

### Ethical aspects

The protocol of the present study was approved by the Research Ethics Committee of the *Universidade Federal de Santa Maria* under CAEE number 02246818.2.0000.5346, opinion 3.022.147 of November 14, 2018. All subjects signed a free and informed consent form, and all the precepts of resolution 466/12 of the Brazilian National Council of Health of the Ministry of Health were followed.

## RESULTS

The study consisted of 36 individuals (18 eutrophic and 18 with overweight), with a mean age of 37.5 ± 10.2 years, and mostly female (80.6%). Most of the samples consisted of individuals with low cardiovascular risk (86.1%). Regarding social status, most individuals were single (52.8%), employed with formal contracts (44.4%), and with over twelve years of education (88.9%). When comparing the sociodemographic characteristics of the eutrophic and groups with overweight, no statistically significant differences were observed between the groups for age, race, cardiovascular risk, marital status, occupation, and education level (Table 1).

**Table 1:** Sociodemographic characteristics of the sample of adult's eutrophic and with overweight (N = 36)

Variables		Sample Total N (%)	Eutrophic N = 18 N (%)	Overweight N = 18 N (%)	P
Age (years – mean ± SD)		37.5 ± 10.2	34.8 ± 9.3	40,2 ± 10,4	0.113^a^
Gender					0.999^b^
	Female	29 (80.6)	15 (83.3)	14 (77.8)	
Global risk score					0.110^b^
	Low risk	31 (86.1)	17(54.8)	14 (45.2)	
Race/ethnicity					0.999^b^
	White	30 (83.3)	15 (83.3)	15 (83.3)	
	Black	2 (5.6)	1 (5.6)	1 (5.6)	
	Brown	4 (11.4)	2 (11.1)	2 (11.1)	
Marital status					0.738^b^
	Single	19 (52.8)	11(61.1)	8 (44.4)	
	Married	15 (41.7)	6 (33.3)	9 (50)	
	Divorced	2 (5.6)	1(5.6)	1 (5.6)	
Occupation					0.948^b^
	Employed with a formal contract	16 (44.4)	9 (50)	7 (38.9)	
	Employed unregistered	12 (33.3)	6 (33.3)	6 (33.3)	
	Household duties	2 (5.6)	1 (5.6)	1 (5.6)	
	Retired	1 (2.8)	-	1 (5.6)	
	Student	5 (13.9)	2 (11.1)	3 (16.7)	
Educational level					0.603^b^
	4-8 years of study	1 (2.8)	-	1 (5.6)	
	9-12 years of study	3 (8.3)	1 (5.6)	2 (11.1)	
	>12 years of study	32 (88.9)	17 (94.4)	15 (83.3)	

a Student's T-Test;

b Fischer's exact test;

SD: standard deviation.

Table 2 presents the anthropometric parameters, laboratory tests, arterial stiffness, and endothelial function parameters of the eutrophic individuals and with overweight. In the group with overweight, all anthropometric parameters were significantly larger compared to the group with eutrophic, except for the waist-to-hip ratio. Regarding the laboratory tests assessed, only the LDL was significantly higher in the group with overweight. There was no statistical difference between the groups regarding the arterial stiffness parameters and the FMD%.

**Table 2:** Anthropometric, biochemical, and cardiovascular parameters of the sample of adult's eutrophic and with overweight (N = 36)

Variables		Sample Total Mean ± SD	Eutrophic N = 18 Mean ± SD	Overweight N = 18 Mean ± SD	P
Anthropometric parameters					
	BMI (kg/m²)	24.74 ± 2.70	22.48 ± 1.65	27 ± 1.31	<0.001
	Waist circumference (cm)	78.58 ± 7.56	73.97 ± 5.18	83.20 ± 1.46	<0.001
	Hip circumferences (cm)	100.6 ± 6.23	96.73 ± 5.45	104.48 ± 4.28	<0.001
	Neck circumference (cm)	33.91 ± 2.98	32.72 ± 2.59	35.10 ± 2.93	0.015
	Sagittal abdominal diameter (cm)	21.85 ± 3.42	19.88 ± 3.42	23.82 ± 2.61	<0.001
	Waist-hip ratio	0.78 ± 0.07	0.76 ± 0.69	0.79 ± 0.74	0.200
	Waist-to-height ratio	0.48 ± 0.43	0.45 ± 0.03	0.51 ± 0.31	<0.001
Biochemical parameters					
	Total cholesterol (mg/dL)	158.19 ± 43.12	151.06 ± 43.5	165.33 ± 42	0.328
	LDL (mg/dL)	70.17 ± 31.38	59.83 ± 24.02	80.5 ± 35	0.047
	HDL (mg/dL)	70.53 ± 23.17	75.22 ± 25.36	65.83 ± 20.37	0.229
	Triglycerides (mg/dL)	86.39 ± 50.72	80.06 ± 35.74	92.72 ± 14.78	0.462
	Nitric oxide metabolites (umol/L)	254.89± 152.63	208.89 ± 123	300.89 ± 163	0.070
Cardiovascular parameters					
	Central SAP (mmHg)	111.25 ± 10.70	111.12 ± 6.24	111.37 ± 14	0.947
	Central DAP (mmHg)	79.57 ± 7.74	78.46 ± 8.69	80.68 ± 6.73	0.397
	AIx@75 (%)	20.83 ± 8.78	20.54 ± 9.0	21.12 ± 8.79	0.845
	PWV (m/s)	5.74 ± 0.93	5.46 ± 0.82	6.02 ± 0.97	0.070
	FMD (%)	6.26 ± 7.42	5.46 ± 8.60	7.06 ± 6.18	0.501

BMI: body mass index; LDL: low-density lipoprotein; HDL: high-density lipoprotein; SAP: systolic arterial pressure; DAP: diastolic arterial pressure; MAP: mean arterial pressure; AIx@75: Amplification Index corrected relative to a cardiac frequency of 75 beats per minute; PWV: pulse wave velocity; FMD: flow-mediated dilation.

Table 3 shows the correlations between the anthropometric parameters and the amplification index, pulse wave velocity, and flow-mediated dilation percentage of the overall sample. The pulse wave velocity presented a positive and moderate correlation with the WC (r = 0.584; P = 0.001), WHR (r = 0.513; P = 0.001), and WHtR (r = 0.590; P = 0.001), and a positive and low correlation with the NC (r = 0.372; P = 0.013) and SAD (r = 0.356; P = 0.033). No anthropometric parameter presented a correlation with the amplification index (AIx@75) and the flow-mediated dilation percentage (FMD%).

**Table 3:** Correlations between the anthropometric parameters and the amplification index, pulse wave velocity, and flow-mediated dilation percentage of the sample in general (N = 36)

Variables	Amplification Index		Pulse Wave Velocity		% Flow-mediated Dilation	
	r	P	R	P	R	P
Body mass index (kg/m^2^)	0.005	0.975	0.251	0.140	0.102	0.553
Waist circumference (cm)	-0.128	0.458	0.584	0.001	0.123	0.474
Hip circumferences (cm)	0.043	0.898	0.103	0.275	0.012	0.471
Neck circumference (cm)	-0.315	0.898	0.372	0.013	0.111	0.260
Sagittal abdominal diameter (cm)	0.054	0.752	0.356	0.033	-0.016	0.925
Waist-hip ratio	-0.162	0.346	0.513	0.001	0.131	0.448
Waist-to-height ratio	0.115	0.253	0.590	0.001	0.197	0.248

P: Pearson's correlation test; kg: kilogram; m²: square meters; cm: centimeter.

Analyzing the correlations of the anthropometric parameters and the amplification index, we verified that, for eutrophic individuals, NC (r = −0.630; P = 0.005) proved to be inversely and moderately correlated with the AIx@75, while the SAD showed an inverse and low correlation (r = −0.488; P = 0.040). In turn, when analyzing the correlations between the anthropometric parameters and the PWV of the eutrophic individuals and with overweight, we found a significant yet low positive correlation of the WHtR (r = 0.471; P = 0.048) with the PWV for eutrophics and moderate positive correlations of the WC (r = 0.535; P = 0.022), WHR (r = 0.593; P = 0009), and WHtR (r = 0.470; P = 0.049) with the PWV for the individuals with overweight. Only the WHtR presented a correlation with the PWV in both groups (Table 4).

**Table 4:** Correlations between the anthropometric parameters and the amplification index, pulse wave velocity, and flow-mediated dilation percentage per eutrophic and overweight group (N = 36)

	Endothelial function and parameters arterial stiffness			
Variables	Amplification Index			
	Eutrophic		Overweight	
	r	P	r	P
Body mass index (kg/m^2^)	-0.267	0.285	0.200	0.426
Waist circumference (cm)	-0.434	0.072	0.039	0.879
Hip circumference (cm)	-0.286	0.250	0.352	0.153
Neck circumference (cm)	-0.630	0.005	-0.379	0.121
Sagittal abdominal diameter (cm)	-0.488	0.040	0.459	0.055
Waist-hip ratio	-0.211	0.401	-0.127	0.616
Waist-to-height ratio	-0.053	0.835	0.308	0.213
	Pulse Wave Velocity			
	Eutrophic		Overweight	
	r	P	r	P
Body mass index (kg/m^2^)	0.164	0.516	-0.198	0.431
Waist circumference (cm)	0.446	0.064	0.535	0.022
Hip circumference (cm)	0.082	0.748	-0.345	0.160
Neck circumference (cm)	0.092	0.716	0.431	0.074
Sagittal abdominal diameter (cm)	0.230	0.358	0.000	0.999
Waist-hip ratio	0.340	0.167	0.593	0.009
Waist-to-height ratio	0.471	0.048	0.470	0.049
	Flow-mediated Dilation Percentage			
	Eutrophic		Overweight	
	r	P	r	P
Body mass index (kg/m^2^)	-0.021	0.935	-0.119	0.638
Waist circumference (cm)	0.220	0.380	0.306	0.216
Hip circumference (cm)	-0.306	0.217	0.064	0.800
Neck circumference (cm)	-0.227	0.365	0.314	0.204
Sagittal abdominal diameter (cm)	-0.366	0.135	0.171	0.497
Waist-hip ratio	0.004	0.987	0.210	0.402
Waist-to-height ratio	0.211	0.402	-0.006	0.982

P: Pearson's correlation test; kg: kilogram; m²: square meters; cm: centimeter.

## DISCUSSION

This study evaluated different anthropometric measurements and their correlations with endothelial function and arterial stiffness parameters in a sample of eutrophic individuals and with overweight We found a positive and moderate correlation of the WC, WHR, and WHtR with the PWV, as well as a positive and low correlation of the NC and SAD with the PWV. No anthropometric measurements in the general sample were associated significantly with the AIx@75 and the FMD%.

The relationship between abdominal adiposity evaluated in this study through the WC has already been described in the literature as an independent risk factor for CVDs (37). This measurement is of simple execution and practical to evaluate visceral adiposity (2). In our study, the WC was positively correlated with the PWV both in the overall sample and for individuals with overweight, demonstrating that abdominal visceral fat has an important correlation with arterial stiffness which precedes endothelial dysfunction (7). Ratifying our findings, Strasser and cols. (19) also found a correlation between the WC with the PWV after adjustments for age and gender in a sample of eutrophic and adults with obesity. The correlation was also observed between the WC and the PWV measured by carotid-femoral tonometry in 531 healthy young people (20).

We did not find a correlation of the WC with the FMD% or with AIx@75. Corroborating our findings, a cohort highlighted that there was no association of any of the anthropometric parameters investigated (BMI, WC, WHR, and WHtR) with the FMD in over 1,400 healthy firefighters, but the sample in the study also included smokers and individuals with diabetes mellitus (17). In turn, Dass and cols. (15) identified a correlation between the WC and FMD (r = 0.3; P < 0.05) in healthy Afro-American males, yet not in women. Another study followed 521 community-based subjects without a history of cardiovascular events for a mean of 8.5 years and who had their measurements of anthropometric and endothelial function. There were long-term increases in weight, waist circumference, and body fat percentage, and these parameters were associated with progressive worsening of microvascular endothelial function, but not conduit vessel endothelial function (38). Since we only analyzed conduit vessel endothelial function (FMD), this may corroborate our results.

We also found a moderate association of the WHR with the PWV both in the overall sample and the individuals with overweight. However, we did not find any correlation between the WHR with the AIx@75 and the FMD. Although the WC is considered a great marker of abdominal fat, the WHR encompasses the distribution of the abdominal fat, which may increase its prediction for the occurrence of arterial stiffness (39). Ratifying our results, Van Den Munckhof and cols. (21) also demonstrated a positive correlation of the WHR with the PWV both in men and women. In ELSA-Brasil the WHR was the one with the best individual performance to estimate the coronary risk (22).

The WHtR is considered an alternative measurement for central obesity, given that it circumvents the limitations of the WC due to the inclusion of height in the index (40). The result of meta-analyses supports the WHtR as an indicator superior to the BMI to identify adults with high cardiometabolic risk, both in men and women (41). In our study, the WHtR proved to be a good anthropometric index associated with arterial stiffness. When evaluating over 1,500 healthy individuals (aged between 50 and 70), Van Den Munckhof and cols. (21) found a strong correlation between the WHtR and the PWV, yet only for male individuals.

SAD has been recommended as an indicator of visceral abdominal fat build-up and cardiovascular risk assessment in recent years (42). Our study demonstrated a positive correlation between the SAD and the PWV in the total sample and a negative correlation with the AIx@75 in the eutrophic group. Our hypothesis holds that the more considerable the atherogenic visceral fat build-up is, the lower the artery relaxation will be given that the AIx@75 corresponds to the reflection intensity of the pulse wave, and the greater the arterial stiffness will be due to the increase in PWV. To the best of our knowledge, besides our study, only Dahlén and cols. (43) have investigated, in 255 individuals with type-2 diabetes and aged between 55 and 66, the association of SAD with arterial stiffness (PWV) also demonstrating a significant result. No previous studies were found analyzing the correlation of SAD with the AIx@75 and FMD.

Another measurement that has been associated with the occurrence of CVD is the NC, which corresponds to a unique fat deposit and may cause a greater predisposition to metabolic risk factors (44). Studies have demonstrated that the NC correlated positively with abdominal visceral fat (45), metabolic syndrome (46) epicardial fat (47), and atherosclerosis (48). Our findings demonstrated a significant positive correlation between the NC with the PWV in the overall sample. Corroborating this, Fantin and cols. (49), when studying healthy individuals affected by overweight and obesity, as well as individuals with hypertension, diabetes, and smoking, found an association between the NC and carotid-femoral PWV.

Regarding BMI, our study did not find any significant association with arterial stiffness or with FMD, demonstrating that BMI is a weak early marker of arterial stiffness and endothelial dysfunction of eutrophic individuals and with overweight healthy at low to intermediate global cardiovascular risk. Although this measurement is effective as an indicator of the nutritional state and a predictor of morbidities in epidemiological studies and even of mortality (3), it presents some limitations, especially the fact that it does not distinguish the body compartments and their distribution (12). Phillips and cols. (18) demonstrated there was no statistical difference between the BMI with the FMD and the PWV in a population of young women with no morbidities, corroborating the results of our study. Other cross-sectional studies with healthy adult individuals also failed to demonstrate associations between BMI and FMD (13,15). In contrast, Williams and cols. (14) evinced in their population with eutrophic and adult individuals with obesity, with a mean age of 36 that the FMD correlated significantly with the BMI (r = 0.3; P < 0.05), although this correlation may be classified as low (35). Another study demonstrated that individuals with metabolic syndrome have an unfavorable profile for FMD, while individuals without metabolic syndrome, either with obesity or not, have a comparable endothelial function. (50). In individuals with obesity and overweight there is an increase in insulin levels and insulin resistance, which promotes vasodilation due to stimulation of nitric oxide (NO) release from endothelial cells. Insulin-induced NO production from vascular endothelium leads to increased blood flow that further enhances glucose uptake in skeletal muscle. This may explain, at least partially, the normal FMD values in this population (51).

In our study, we also found no association of BMI with the AIx@75. Backing our findings, Solanki and cols. (52), upon comparing the BMI with the AIx@75 in a healthy population aged between 15 and 35 also assessed by the oscillometric technique, did not find a correlation. Similar results were described by Wykretowicz and cols. (53), in adult individuals with no previously established diseases, in which the BMI did not correlate (r = 0.09; P = 0.22) with the AIx@75 by tonometric assessment.

Although our findings were consistent with those described in the previously mentioned studies, other works (16,21) demonstrated significant results of the BMI with the PWV and AIx@75. In a cohort that was prospectively followed since birth in a southern Brazilian city demonstrated that the BMI, visceral adipose tissue thickness and fat mass were the strongest predictors positively correlated with PWV at 30 years of age (54). However, the population in such studies included not only healthy adults, but also the elderly, smokers, and individuals with comorbidities (hypertension and diabetes mellitus). Nabeel and cols. demonstrated that PWV increases with age and with risk factors for metabolic syndrome. (55).

Our study demonstrated that the WC, WHR, and WHtR, were good anthropometrics parameters correlated with arterial stiffness. Although less used in clinical practice and despite the scarcity of studies, the SAD and NC also demonstrated correlations, albeit weaker, with the arterial stiffness parameters. From the clinical viewpoint, our study emphasizes the importance of using simple anthropometric measurements as additional tools to identify individuals at cardiovascular risk.

Despite the results, it is worth stressing that our study has several limitations. First, it's characterized as cross-sectional, which does not allow for establishing a cause-effect relationship, thus evincing the need for more prospective and controlled studies. Second, the sample size was small, thus insufficient statistical power must be acknowledged. Finally, we included only young and middle-aged individuals, the majority with low cardiovascular risk, a population whose endothelial function still could be preserved.

In conclusion, the present study demonstrated significant correlations between the WC, WHR, WHtR, SAD, and NC with the PWV in the overall sample, but not with the AIx@75 and FMD. Our findings emphasize the importance of applying simple anthropometric measurements in clinical practice to identify individuals at cardiovascular risk.
